# Data-driven systems to detect physical weakening from daily routine: A pilot study on elderly over 80 years old

**DOI:** 10.1371/journal.pone.0274306

**Published:** 2023-01-30

**Authors:** Manuel Abbas, Majd Saleh, Dominique Somme, Régine Le Bouquin Jeannès

**Affiliations:** 1 Univ Rennes, Inserm, LTSI UMR 1099, Rennes, France; 2 Univ Rennes, CNRS, ARENES UMR6051, Rennes, France; Universita degli Studi di Milano, ITALY

## Abstract

The use of telemonitoring solutions via wearable sensors is believed to play a major role in the prevention and therapy of physical weakening in older adults. Despite the various studies found in the literature, some elements are still not well addressed, such as the study cohort, the experimental protocol, the type of research design, as well as the relevant features in this context. To this end, the objective of this pilot study was to investigate the efficacy of data-driven systems to characterize older individuals over 80 years of age with impaired physical function, during their daily routine and under unsupervised conditions. We propose a fully automated process which extracts a set of heterogeneous time-domain features from 24-hour files of acceleration and barometric data. After being statistically tested, the most discriminant features fed a group of machine learning classifiers to distinguish frail from non-frail subjects, achieving an accuracy up to 93.51%. Our analysis, conducted over 570 days of recordings, shows that a longitudinal study is important while using the proposed features, in order to ensure a highly specific diagnosis. This work may serve as a basis for the paradigm of future monitoring systems.

## Introduction

With rising life expectancy, our society is getting older, and the majority of older adults would like to age at home. Nonetheless, frailty which leads to progressive physical weakening and increases the risk of falls and lack of autonomy [1, 2], threatens this lifestyle choice. This geriatric syndrome has been a trending topic in the past decades, and remains an emerging clinical and public health priority [3, 4]. Even though its definition is controversial [5], frailty is conceptualized as a clinical syndrome symbolized by reduced resistance to stressors, resulting from age-associated cumulative declines in physiological function across multiple organ systems [6, 7]. It is mainly identified by functional weakening and sarcopenia (*i.e.* loss of muscle tissue). Hence, the physical function is one of the most decisive elements in the prevention and therapy of old people’s health conditions [8, 9].

Fried *et al.* proposed the frailty phenotype (FrP) which is based on five criteria [10] and is one of the widely-used clinical tools for screening. Some other self-reported measures and performance tests like the Short Physical Performance Battery (SPPB) [11] and the International Physical Activity Questionnaire (IPAQ) [12] were also considered to evaluate mobility and to estimate the level of physical activity. While this kind of approaches is helpful in well-being assessment, it is not sufficient to define a full and accurate tracking system, seeing that most of these measurements (i) are subjective, (ii) are calculated under supervised conditions, and (iii) provide short-term estimation of physical capacities. This justifies the importance of telemonitoring solutions for elderly care. More recently, some works conducted observational studies by monitoring a set of different activity metrics from wearable sensors in relation to frailty status and health conditions. A pendant sensor was employed to monitor the physical activity of elderly (≥ 60 years old) over 48 hours, by observing the body posture (walking, sitting, standing), the activity behavior (sedentary, light, moderate-to-vigorous), steps parameters, and sleep parameters [13]. This study was further extended to assess cognitive frailty in community-dwelling older people [14]. The goal was to recognize the presence of both (i) physical frailty (defined by FrP) and (ii) cognitive impairment (defined by the score of Mini-Mental State Examination) using a chest-worn sensor over 48 hours. Another research has taken place to evaluate the reliability of wearable devices to assess frailty in older home care clients [15]. Acquired data of participants aged 55 years and older were exploited to derive variables including daily step count, sleep time and quality, and heart rate over a period of 9.43 (± 1.99) days. Besides, gait parameters, such as gait speed, cadence and stride time were inspected to assess frailty conditions [16]. Those parameters were calculated under supervised conditions, where participants walked a distance of 4 m at the self-selected usual pace, using wireless sensors fixed on the lower limb segments. Although the importance of remote monitoring was demonstrated in these works, we can identify several limitation in the literature. Firstly, wider sets of extracted variables are required. For instance, the muscle strength, the reduction of leisure activities (LAs), and instrumental activities of daily living (iADLs) are mainly associated to the functional capabilities limitations [17]. Therefore, it is important to observe the number of times an older person quits his/her house by taking the lift/stairs, a parameter that has not really been studied in the literature. In addition, researchers have yet to determine which indicators are mostly useful to quantify physical activity of elderly [18]. To date, the most relevant/discriminant features are not well-known, and whether they are subject-dependent is still an open question. Secondly, most experiments include relatively young volunteers (less than 65 years old) [13, 15]. To the best of our knowledge, recruiting octogenarians and older ones and analyzing their data have received little, if any, attention in the literature. Thirdly, numerous studies were based on clinical trials under supervised conditions with a predefined experimental setup [16, 19, 20]. This might lead to biased results seeing that the experiments, where the subject is instructed to perform a set of movements, do not reflect his real behavior in free-living conditions. Fourthly, a short-term evaluation is usually considered [13, 14, 21] (24–48 h to few days) while monitoring the subjects. The short-term experiments may be insufficient to represent the overall activities of elderly in natural conditions. More importantly, the research design (cross-sectional *vs* longitudinal study) in this context has not been a topic of discussion.

Accordingly, this paper presents a pilot study conducted on elderly over 80 years of age. The objective of this work is to understand what happens to the physical function during the transition towards frailty in terms of acquired data, and see which activity metrics are able to identify the commonalities between seniors of diverse profiles. Consequently, we propose a data-driven system, by developing a fully automated monitoring process which computes activity measurements using a single wearable device (placed on the body trunk) to detect physical weakening and distinguish healthy elderly from frail ones. This work takes into consideration the limitations of the literature to address the aforementioned ambiguities. Explicitly, our contributions are fourfold:

- an ensemble of heterogeneous features is proposed to characterize the physical function of the individual.
- the proposed variables are computed over more than 1.5 year of recordings during the daily routine of older adults. Hence, our work ensures a long-term evaluation in natural and unsupervised conditions, contrarily to the majority of literature studies where relatively younger individuals are recruited and monitored over a short period, whose actions are limited to a given set of instructions.
- the statistical significance of the proposed features, as well as their discrimination power using several classifiers are evaluated to see if they are able to separate both populations or if they are related to each individual, a question that has not been well addressed in the literature.
- the efficacy of predictive models is assessed following two setups (thanks to the long-term experimental observation) to reveal the best research design, namely (i) daily assessment *vs* (ii) longitudinal assessment.

## Materials and methods

This work involved human subjects in its research. Approval of all ethical, experimental procedures, protocols, and use of data was granted by the Ethics Committee of the University Hospital of Rennes, France under Approval No. 19.56 and the Ouest VI Institutional Review Board of Morvan University Hospital of Brest, France under Approval No. 1228.

### Instrumentation & preprocessing

**Fig 1:**
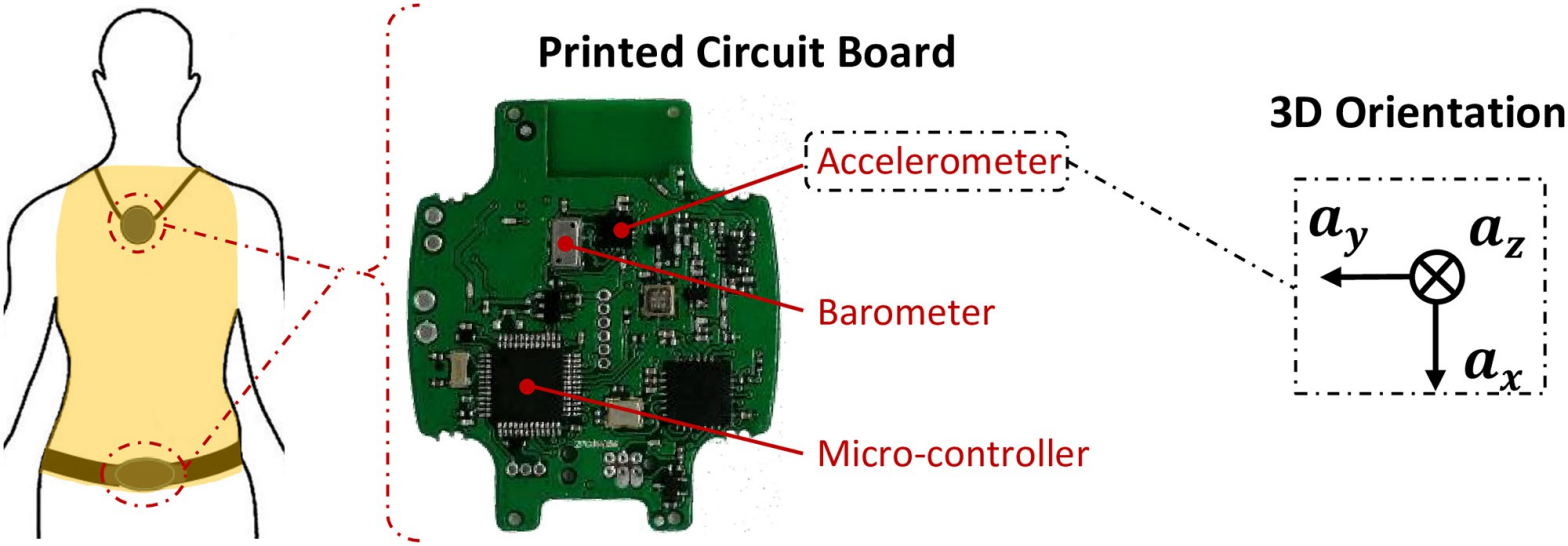
Wearable device. The sensing unit placement (body trunk in yellow), with the corresponding printed circuit board and the 3D orientation of the accelerometer.

The wearable device designed for this study was developed by our partner company RF-Track (Cesson-Sévigné, France). It consists of two sensors, namely a tri-axial accelerometer and a barometer. This choice was based on our previous studies, in which it has been shown that acceleration signals alone are sufficient for an accurate identification of physical activities and body postures [22, 23]. The barometer was added to estimate the device altitude, an added-value to the inertial system. Fig 1 illustrates the corresponding printed circuit board (PCB) and its elements. The employed Micro-Electro-Mechanical System (MEMS) 3D accelerometer is LIS3DH from STMicroelectronics, measuring {*a*_*X*_, *a*_*Y*_, *a*_*Z*_}. It was configured to a sampling frequency of ^*A*^*F*_*s*_ = 50 Hz with a measurement range of ±8 g. MS5611–01BA03 from TE Connectivity is the barometric pressure sensor, measuring both the atmospheric pressure *P* and the temperature *T* with a sampling frequency of ^*B*^*F*_*s*_ = 5 Hz. Raw data of both sensors are preprocessed by the ultra-low-power micro-controller STM32L431 from STMicroelectronics. Explicitly, the acceleration magnitude ||*a*|| is computed as follows:

$$\begin{array}{c} \left||a|\right|=\sqrt{a_{X}^{2}+a_{Y}^{2}+a_{Z}^{2}} \end{array}$$ (1)

Moreover, sudden peaks are filtered out from *P* using a slopeLimit filter [24]. Afterwards, the altitude *H* is calculated using the temperature *T* and the filtered pressure *P*′ as in [25]:

$$\begin{array}{c} H=\frac{T}{0.0065}\times [1-{\left(\frac{P^{\prime }}{1013.25}\right)}^{0.19}] \end{array}$$ (2)

where *H* is in meters, *T* in kelvin, and *P*′ in mbar. The PCB was integrated inside an enclosure, which can be placed on the body trunk (see yellow zone in Fig 1), as a pendant or around the waist. This placement (body trunk) is close to the center of body mass, which increases the reliability of the processing units. Moreover, it was seen in a previous work that a single algorithm can cover both positions [22] since the movements captured by those devices are close. This is important since the senior can choose the position that suits him best, increasing the acceptability of the monitoring system.

### Feature extraction from raw data

We now describe the extraction process of nine statistical and time-domain features characterizing the physical function of the device wearer. This approach scans 24 hour-file (input), consisting of {*a*_*X*_, *a*_*Y*_, *a*_*Z*_}, *P* and *T*. The date and time during which the subject has worn the device are also indicated. The proposed features *G*_*i*_ are detailed hereafter.

#### Activity rate

||*a*|| is divided into *K* 30-second windows. For each window *i*, the magnitude is demeaned, and the root mean square (rms) is computed, measuring the varying quantity in the signal. If its value exceeds a certain threshold, for example 3%, the window is labeled as active, meaning that the subject is moving. Otherwise, the subject is considered as inactive/quiet. Suppose *K*_*A*_ the number of windows labeled as active, thus:

$$\begin{array}{c} G_{1}=\frac{K_{A}}{K} \end{array}$$ (3)

Note that, when the subject is inactive, the orientation of acceleration components (*a*_*X*_, *a*_*Y*_, *a*_*Z*_) is studied to estimate the body posture (sitting or lying).

#### Sleep calmness

The system enters “night mode” between 10:00 pm and 8:00 am (the following day). Throughout this time interval, periods during which the elderly is inactive and lying are detected. A processing unit decomposes the corresponding 3 acceleration components {*a*_*X*_, *a*_*Y*_, *a*_*Z*_} into 5-second fragments with 80% overlapping (4 seconds). We assume that a sleep cycle consists of 5 minutes without any slight movement. The standard deviation (SD) and range values of each component are then calculated. We call silent fragments (where the subject is not moving at all) those for which the three components display low SD values (less than 3% in our case) and low range values (below 0.1 g). Afterwards, windows consisting of consecutive silent fragments are detected, and the corresponding duration *S* is measured. Sleeping cycles are windows where *S* exceeds 5 minutes. The sleeping time (SpT) is equal to the sum of sleeping cycles’ lengths. Lying time (LyT) is the time spent between the first and last cycles while lying. Thus, this second feature *G*_2_, which is a rough estimate of sleep calmness (SC), is computed as:

$$\begin{array}{c} G_{2}=100\times \frac{\text{SpT}}{\text{LyT}} \end{array}$$ (4)

This feature allows us to investigate a potential relation between sleep agitation and physical impairment.

#### Number of steps

**Fig 2:**
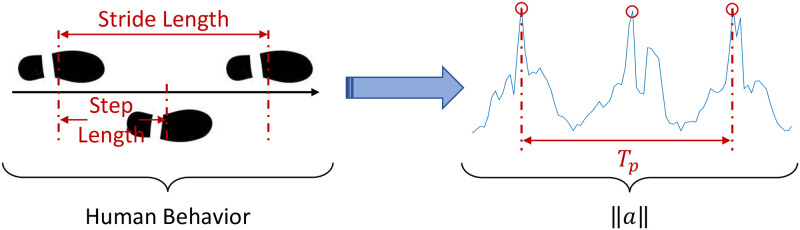
Gait acceleration pattern. The human behavior *vs* the acceleration magnitude ||*a*|| during periodic movements, particularly while walking.

The steps are identified by peaks *pk*_*i*_ in ||*a*|| (Fig 2) when the subject is (a) standing, *i.e.* *a*_*X*_ is vertical (as displayed in Fig 1), and (b) moving (rms > 3%). The peaks *pk*_*i*_ are detected using the acceleration-based method proposed by Abbas *et al.* [26]. The number of steps is estimated by the third feature *G*_3_:

$$\begin{array}{c} G_{3}=\sum_{i=1}^{N}pk_{i} \end{array}$$ (5)

#### Periodicity

The objective of this parameter is to measure the time span of continuous walking/jogging (non-stop). It inspects the capability of elderly to reproduce periodic movements continually over long spells. Accordingly, the auto-correlation sequence of 30-second acceleration ||*a*|| windows *w*_*i*_ (where the elderly is active) is computed. Afterwards, the resultant sequence of *M* peaks $P_{i}$, whose values exceed 0.3, are detected and localized. Note that the acceptable peak-to-peak separation $dP_{i}$ is restricted to a minimum of 250 ms. Howbeit, the output of auto-correlation must represent at least 3 peaks in order to consider the movement as periodic. Therefore, two features can be computed, namely *G*_4_ which is the periodicity rate (autocorrelation sequences over 24 h) and *G*_5_ which is the period *T*_*p*_ of the periodic movements. Suppose that T is the number of windows *w*_*i*_ which represent periodic movements, *D* is the monitoring/observation period in seconds, and $dP_{i}$ is the distance (in seconds) between the *i*^th^ and the (*i* + 1)^th^ peaks, hence:

$$\begin{array}{c} G_{4}=100\times \frac{30\times T}{D} \end{array}$$ (6)

and

$$\begin{array}{c} G_{5}=\frac{1}{{}^{A}F_{s}}\times \frac{1}{M-1}\sum_{i=1}^{M-1}dP_{i} \end{array}$$ (7)

Fig 2 symbolizes the relationship between this metric and the real human behavior. The period *T*_*p*_ represents, in a certain way, the stride of human gait. For example, if a subject has a stride of length 80 cm and a period equal to 800 ms, his walking speed is equal to $\frac{0.8}{0.8}=1$ m/s.

#### Weightlessness

This condition is met when the effect of gravity is canceled by inertial force, like in free-fall. For instance, when the subject is jumping or jogging, the human body quits the ground for short spells [27]. On the way back, the value of acceleration magnitude ||*a*|| is close to 0 g (weightlessness interval). The sixth feature *G*_6_, being the length *l*_*i*_ of these intervals, is linked to the subject’s strength and ability of leaving the floor in such high intensity activities:

$$\begin{array}{c} G_{6}=\sum_{i}l_{i} \end{array}$$ (8)

#### Change in altitude

**Fig 3:**
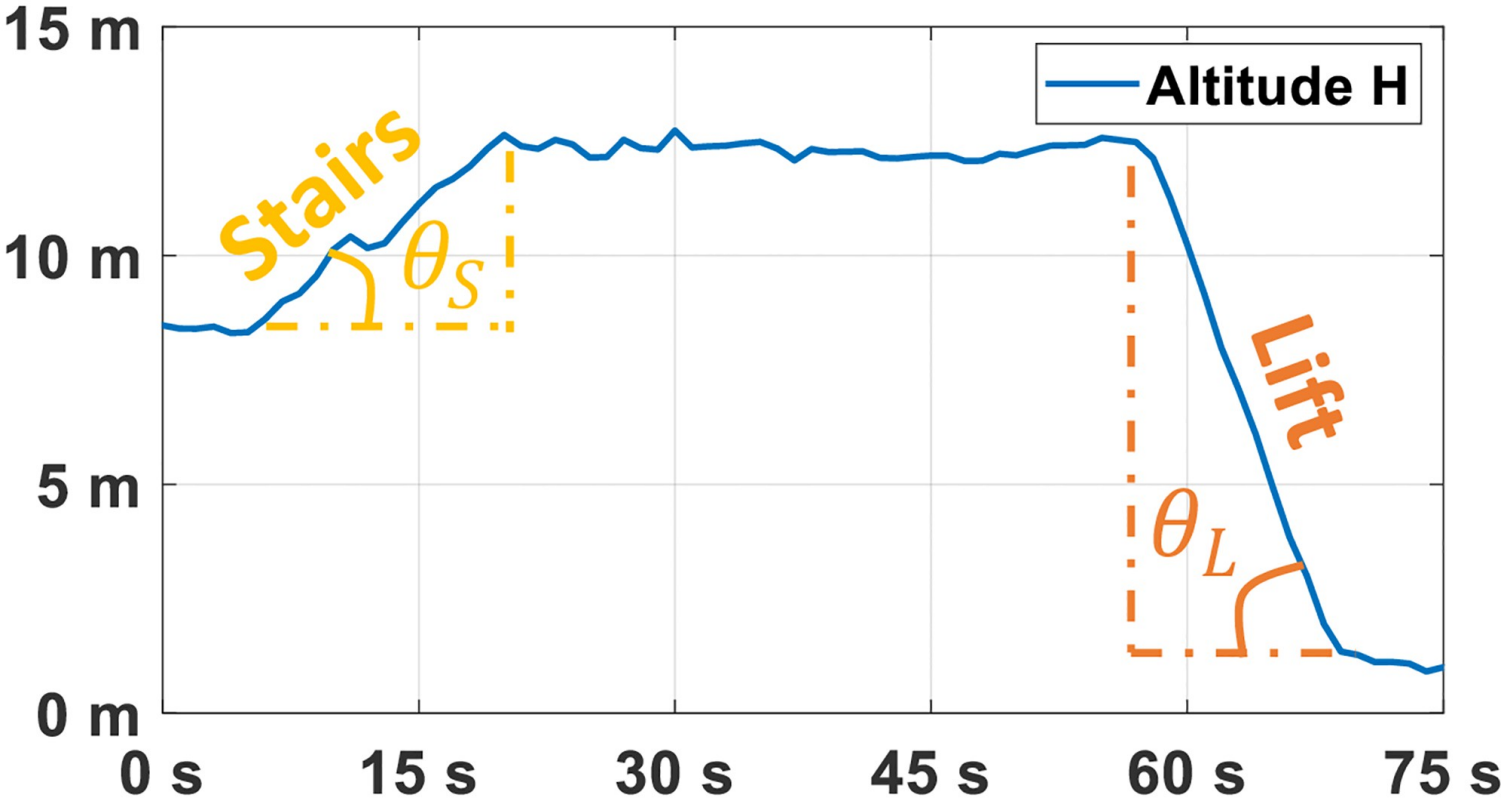
Altitude via barometric signals. The change in altitude while using stairs (tan(*θ*_*S*_) ≈ 0.23*m*/*s*) and while using lifts (tan(*θ*_*L*_) ≈ 0.66*m*/*s*).

A change in atmospheric pressure *P* arises following three scenarios: (i) when we use the lift, (ii) when we use the stairs, (iii) when a sudden change in temperature occurs (while opening the window or moving between two rooms for example). To discriminate between these behaviors, we begin by estimating the altitude of the device at each second, using Eq 2. We then localize periods where the altitude is increasing or decreasing continuously (for at least 6 s). Next, we measure the slope tan(*θ*) of the ascending/descending curve, the resultant change in height Δ*H*, and the rms of ||*a*|| (to see if the subject is static/moving). Fig 3 illustrates the evolution of time-series representing the altitude of the device when the subject uses the stairs then the lift.

Therefore, when the subject is inactive or slightly moving and the slope is varying considerably (in the range of 0.6–0.9 m/s for example), the corresponding interval is labeled as “lift” and the variable *n*_*L*_, originally set to 0, is incremented by 1. Meanwhile, when the subject is active and the altitude is rising/decreasing moderately (0.25 ≤ |tan(*θ*)| ≤ 0.6), the corresponding interval is labeled as “climbing/descending stairs” and the variable *n*_*S*_, originally set to 0, is incremented by 1. Consequently, two features *G*_7_ and *G*_8_ are considered based on the change in altitude (CIA), estimating the number of times the subject has used the lift and the stairs respectively:

$$\begin{array}{c} G_{7}=n_{L} \end{array}$$ (9)

and

$$\begin{array}{c} G_{8}=n_{S} \end{array}$$ (10)

#### Energy expenditure

The metabolic equivalent of task (MET) is a kind of proportionality constant resulting from the relationship between the intensity of physical activity and energy expenditure (EE). Based on this constant, one may calculate the number of burned calories:

$$\begin{array}{c} \text{Burned}\;{\text{Calories}}_{\left(\text{kcal}\right)}=\text{MET}\times {\text{Duration}}_{\left(\text{h}\right)}\times {\text{Weight}}_{\left(\text{kg}\right)} \end{array}$$ (11)

Haskell *et al.* discussed the MET values of some activities [28], depending on their intensity. In our approach, the same logic is followed. Based on the type and the intensity of the movements, a MET value is assigned using Table 1. For instance, the inactivity periods, where the MET is equal to 1, are localized as explained previously. Besides, when the subject is active, the type of the movements (periodic/irregular), its intensity (which is proportional to the SD value of the acceleration magnitude *σ*_||*a*||_), and the corresponding time-span (duration) are known. When the subject is slightly moving (light movements such as stand-to-walk for example), the corresponding MET is equal to 1.5 if *σ*_||*a*||_ is less than 0.025, and to 1.8 otherwise. For cyclic activity, when the subject is walking slowly (low intensity periodic movements, *σ*_||*a*||_ < 0.09), the corresponding MET is equal to 2, whereas when he is walking briskly (*σ*_||*a*||_ > 0.2), the MET is equal to 5. Meanwhile, when the subject is walking moderately (*i.e.* 0.09 < *σ*_||*a*||_ < 0.2), the MET is equal to 3.3 (see Table 1). Let us indicate that MET values greater than 6 are linked to very high intensity movements like jumping or jogging where the weightlessness state occurs. Therefore, the number of burned calories $C_{i}$ resulting from the *i*^th^ window is estimated using Eq (11), and the ninth feature *G*_9_ is an approximation of EE over the course of the day, and is computed as:

$$\begin{array}{c} G_{9}=\sum_{i}C_{i} \end{array}$$ (12)

**Table 1:** MET equivalents depending on activity class and intensity.

State	Inactive	Active				
Type & Intensity	—	Light Movements	Periodic			Weightlessness
			Low	Moderate	Brisk	
**MET**	1	1.5 - 1.8	2	3.3	5	> 6

### Study cohort and experimental setup

To evaluate the efficacy of the proposed features, 18 older adults over 80 years old, who provided their informed consent, were recruited from June 2019 to February 2021. Recruitment was based on two distinct populations. On the one hand, vulnerable participants, who have fallen at least once in the previous year, were recruited during a study involving nursing home permanent resident. On the other hand, the second population includes non-frail community-dwelling elderly, who have no frailty criteria according to FrP, and who were recruited by press announcements, posters placed in medical offices (notably geriatrics consultation) and by communication in retired-people association or housing facilities. The study population was perfectly balanced (50% frail subjects). On average, participants were monitored for 31.6 (± 16.5) days, leading to a total of 570-day recordings. These subjects wore the sensing device during their daily routine without any specific instruction being imposed. The corresponding acceleration and barometric data were recorded on a memory card. The research team intervened to download data and format the memory card. Table 2 illustrates some relevant demographic details of the study cohort.

**Table 2:** Demographic details of the study cohort.

Age	Height_(m)_	Weight_(Kg)_	Distribution
[80, 92]^†^	[1.5, 1.71] (1.6 ±0.06)^‡^	[52, 84.6] (63.2 ±9.2)	50% robust *vs* 50% frail

^†^ range—

^‡^(mean ± SD)

## Results

### Outcome in free-living conditions

The proposed features have been extracted from data collected in real world conditions to see whether they are able to identify people showing signs of impaired physical conditions or not. The computed measurements were stored in a 2D array *F* of size 570 × 9, each column representing a feature *G*_*i*_. They subsequently were averaged per subject, resulting in 18 values for each metric (9 frail vs 9 robust), thus a matrix *F*′ of size 18 × 9. Afterwards, the mean, SD, lower and upper quartile (*Q*_1_ & *Q*_3_) of these 18 values are calculated. Table 3 illustrates the corresponding results.

**Table 3:** Different statistics representing the extracted health measurements of (a) frail people and (b) healthy (non-frail) old people.

	Features	Steps	Activity Rate*	Periodicity		CIA^†^^†^		SC*	EE^‡^
				Rate*	*T_p_* ^†^	Lift	Stairs		
**Frail**	mean (±SD)	1019 (±939)	14.5 (±4.8)	0.23 (±0.3)	1.9 (±0.63)	0.98 (±0.92)	0.02 (±0.05)	75.4 (±10.5)	19.7 (±2)
	$\left(\begin{array}{c} Q_{1}\\ Q_{3} \end{array}\right)$	$\left(\begin{array}{c} 397\\ 1895 \end{array}\right)$	$\left(\begin{array}{c} 11.5\\ 18.9 \end{array}\right)$	$\left(\begin{array}{c} 0\\ 0.45 \end{array}\right)$	$\left(\begin{array}{c} 1.5\\ 2.25 \end{array}\right)$	$\left(\begin{array}{c} 0.05\\ 1.38 \end{array}\right)$	$\left(\begin{array}{c} 0\\ 0.04 \end{array}\right)$	$\left(\begin{array}{c} 74.8\\ 80.6 \end{array}\right)$	$\left(\begin{array}{c} 18.3\\ 21.1 \end{array}\right)$
**Healthy**	mean (±SD)	4663 (±2036)	20 (±5.7)	3.5 (±5)	1.18 (±0.04)	1.58 (±1.7)	1.41 (±1.12)	88.2 (±10.7)	26.6 (±2.3)
	$\left(\begin{array}{c} Q_{1}\\ Q_{3} \end{array}\right)$	$\left(\begin{array}{c} 2819\\ 6658 \end{array}\right)$	$\left(\begin{array}{c} 14.9\\ 23.5 \end{array}\right)$	$\left(\begin{array}{c} 1.2\\ 2.6 \end{array}\right)$	$\left(\begin{array}{c} 1.14\\ 1.22 \end{array}\right)$	$\left(\begin{array}{c} 0.46\\ 2.64 \end{array}\right)$	$\left(\begin{array}{c} 0.8\\ 1.8 \end{array}\right)$	$\left(\begin{array}{c} 76.5\\ 96.6 \end{array}\right)$	$\left(\begin{array}{c} 24.4\\ 28.5 \end{array}\right)$

* Rates are in %—

^†^ *T*_*p*_ is in seconds—

^†^^†^ CIA is in times/day—

^‡^ EE is in kcal/kg

Based on these values, one can see that both populations are globally distinguishable with respect to the proposed features. Firstly, robust people are more dynamic, since on average, their number of steps taken is around 4.5 times that of frail ones (4663 *vs* 1019) and their activity rate is significantly higher. Additionally, the normalized energy expenditure (EE) decreases when people become frail, since they are less energetic and their activities involve lower intensity. Secondly, periodicity is hardly detectable in acquired signals of frail people. Besides, the period of this type of movements *T*_*p*_, is significantly higher for this population compared to healthy elderly. Thirdly, it is apparent that frail older individuals do not take the stairs, but some few outliers do exist (∼ 0.02 times per day). Finally, the sleep pattern seems to be a bit quieter for healthier subjects. Let us indicate that the weightlessness state is non-existent for both groups, thus *G*_6_ is not included in the table.

**Fig 4:**
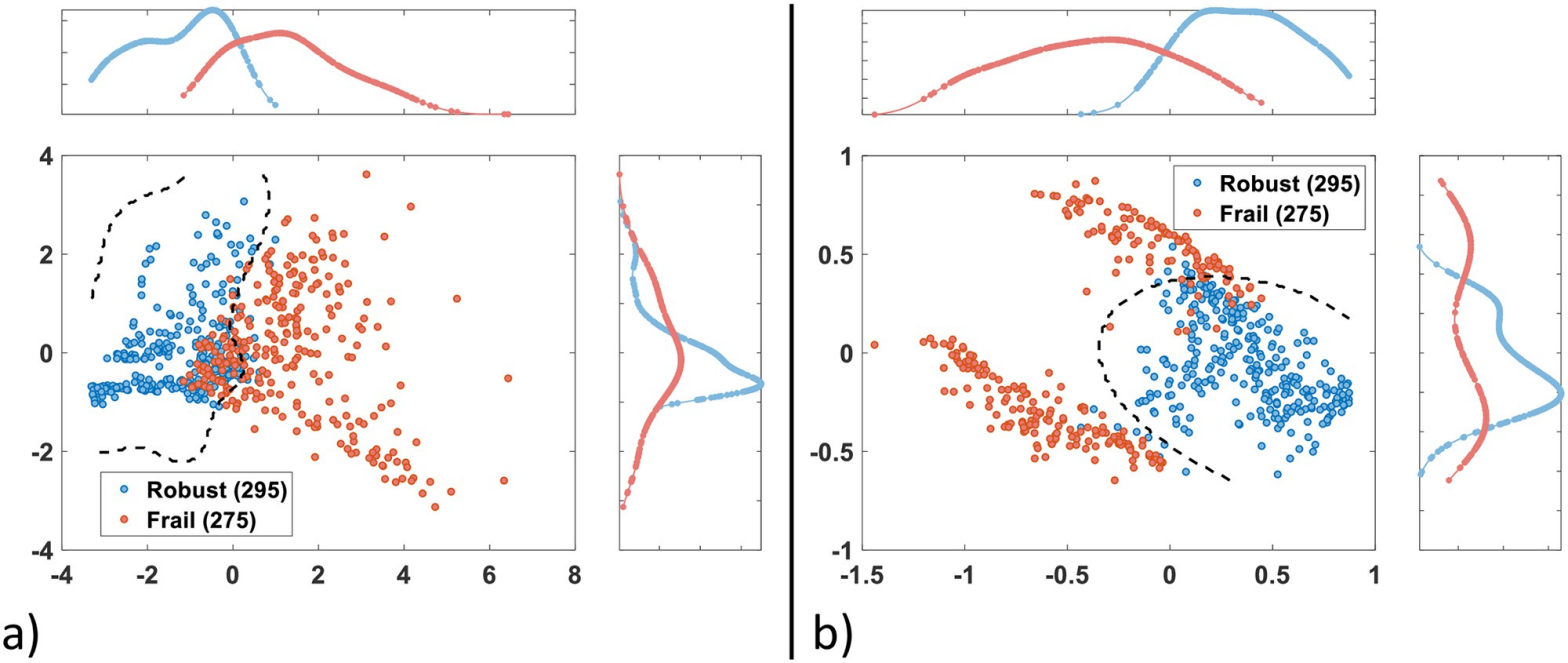
Principal component analysis (2D). Data distribution of both populations following two principal components, with the SVM boundary in dashed lines, after being scaled using (a) z-score formula and (b) sigmoidal function.

Table 3 defines both populations from wearable sensors point of view. Nonetheless, it does not provide a concrete view regarding data distribution in the space. Consequently, the feature vectors constituting *F* were then scaled since they vary in magnitude, range, and unit. Two models were used, (i) a linear model, namely z-score (Eq 13), (ii) a non-linear model, namely sigmoid (Eq 14):

$$\begin{array}{c} \hat{f_{i}}=\frac{f_{i}-\mu }{\sigma } \end{array}$$ (13)

$$\begin{array}{c} \hat{f_{i}}=\frac{1}{1+exp(-\frac{f_{i}-\mu }{\sigma })} \end{array}$$ (14)

with $\hat{f_{i}}$ an element in the scaled vector (a column of $\hat{F}$), *f*_*i*_ an element in the original vector (a column of *F*), *μ* and *σ* the mean value and SD of the original vector respectively. Afterwards, principal component analysis (PCA) was applied on each of the two scaled datasets to reduce the dimensionality by minimizing information loss, thus to increase the interpretability. This reduction allows us to visualize data in a 2-dimensional space in order to observe trends, jumps, clusters and outliers. Data of both populations were then plotted in a 2D space following two principal components (PCs), as illustrated in Fig 4. Each point represents a day. Additionally, a kernel smoothed density was estimated and plotted for each population (top and side distributions) using the scores of the PCs. Two observations can be noted. Firstly, using sigmoid increases the discrimination power of the classification system. As seen in Fig 4a (z-score), a huge overlap occurs between the side distributions (PC-2). Secondly, both populations are non-linearly separable. To confirm these observations, the boundary of Support Vector Machine (SVM) with Radial Basis Function (RBF) kernel is shown in both plots (dashed line). After being cross validated, the two feature vectors (PCs) fed the aforementioned classifier. The achieved balanced accuracy was 89.53% when using z-score and 96% when using sigmoid. It is worth mentioning that the aforementioned result is not meant to represent the discrimination power of the features. It is just reported to show the superiority of sigmoidal model when it comes to feature scaling. Hence, for the next subsection, the feature vectors are scaled using Eq 14.

### Relevant features identification & classification process

**Fig 5:**
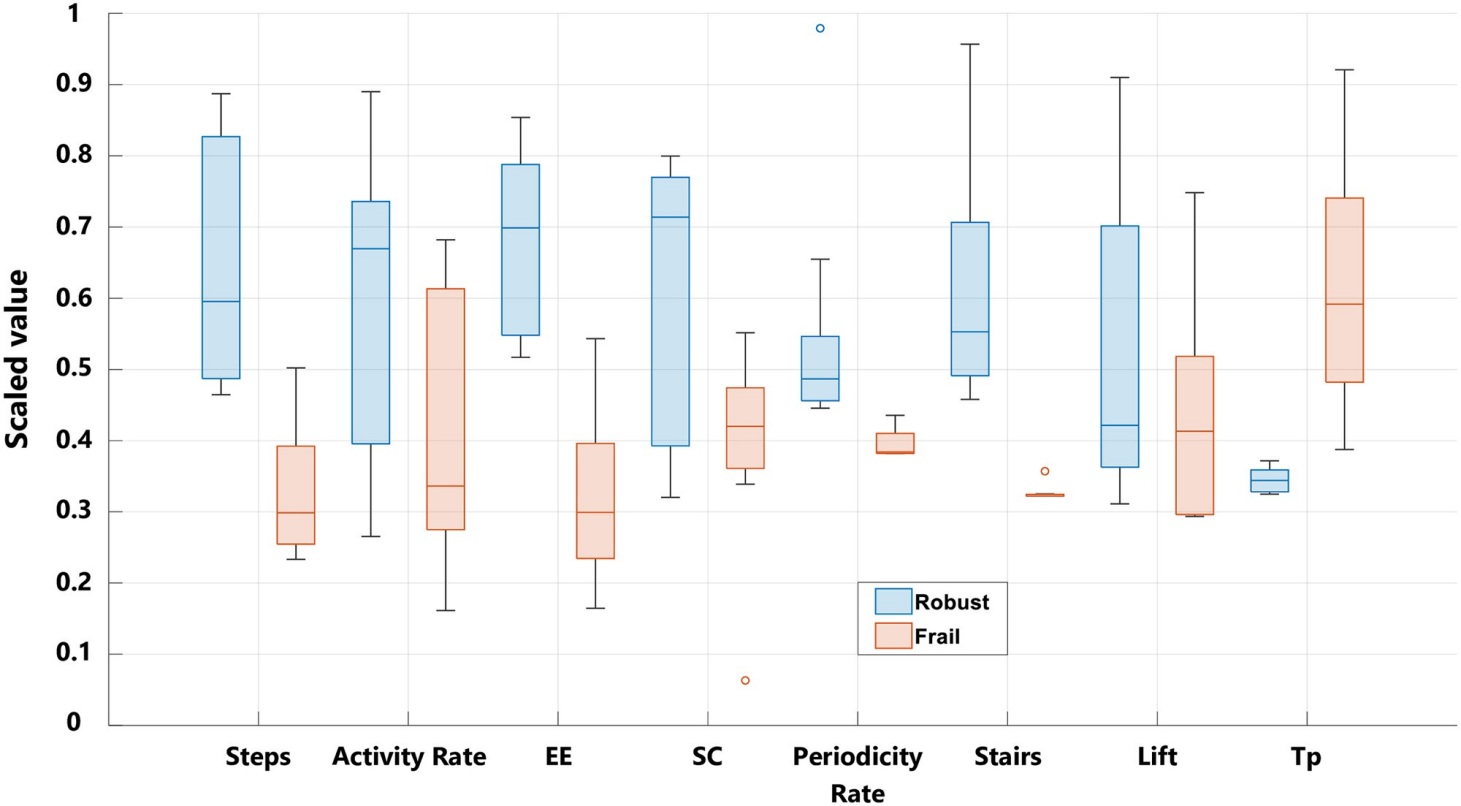
Box charts of extracted features. The set of box charts showing the data distribution of each feature (x-axis) following both populations (Robust *vs* Frail).

Although PCA reduced the dimensionality and transformed the set of heterogeneous variables into a smaller one (2D) that still carries most of the information in the larger set, it does not reveal the individual performance of each feature, an important topic from a clinical point of view. Fig 5 displays the box plot for each extracted feature (after being scaled). The two box plots are well separated when it comes to the number of steps, the EE, the periodicity rate, the frequency of using the stairs, and the period *T*_*p*_. Even though the median of robust people is higher when it comes to Activity Rate and SC, there is still a significant intersection between both populations. Moreover, using the lift is irrelevant to the discrimination between the two groups of elderly. To confirm this observation, and in order to identify statistically significant features, we ran the Wilcoxon rank sum test on *F*′. Table 4 illustrates the results in terms of p-value. These results (at a 5% significance level) are coherent with the box charts. The most relevant features in this context are the number of steps, the EE, the periodicity rate, the frequency of using the stairs, and *T*_*p*_ with p < 0.05. Therefore, these features are retained from $\hat{F}$ to feed machine learning classifiers, constituting a matrix $\tilde{F}$ of size 570 × 5.

**Table 4:** The Wilcoxon signed rank test results for each metric in terms of p-value.

Measurement	Steps	Activity Rate	EE	SC	Periodicity	Stairs	Lift	*T*_*p*_
**p-Value**	**<8 × 10^−4^**	0.0625	**<2 × 10^−4^**	0.077	**<5 × 10^−5^**	**<5 × 10^−5^**	0.128	**<5 × 10^−5^**

The combination of these five features is now tested to identify subjects with physical weakening. Being frail is considered the positive class. The feature vectors of $\tilde{F}$ fed five different classifiers, namely Neural Network (NN) of 1 hidden layer of 15 neurons with a sigmoidal activation function, SVM with RBF kernel, *k*-Nearest Neighbors (*k*-NN) with *k*=5, Random Forest (RF) of 10 estimators and a maximum depth equal to 5, and Gradient Boosting Machine (GBM) of 100 estimators with a learning rate of 0.4. Leave-Subject-Out (LSO) cross validation was applied, *i.e.* the classifier was trained on 17 subjects, then tested on the remaining one 18 times. The overall positives and negatives of these predictions were counted to evaluate the sensitivity, specificity, and accuracy using true positive (TP), false positive (FP), true negative (TN), and false negative (FN) values:


$$\begin{array}{c} \left\{ \begin{array}{c} \text{specificity}=\frac{\text{TN}}{\text{TN}+\text{FP}}\\ \text{sensitivity}=\frac{\text{TP}}{\text{TP}+\text{FN}}\\ \text{accuracy}=\frac{\text{TN}+\text{TP}}{\text{TN}+\text{TP}+\text{FN}+\text{FP}} \end{array}\right. \end{array}$$

Table 5 illustrates the results of the first research design, namely a daily prediction. NN achieves the highest accuracy (93.51%) and the highest sensitivity (92.88%), which is the ability of a classifier to correctly identify frail people with a damaged physical function. GBM has the lowest false positive rate (4.73%). Hence, if we want to notify the medical staff whether the subject is becoming frail or not (on a daily basis), in order to intervene for a clinical examination, the system will send around one false alarm every 3 weeks using GBM. However, GBM is less sensitive than NN. RF is a close competitor to GBM, but with lesser specificity and sensitivity. The specificity/sensitivity trade-off should be found depending on the medical diagnosis.

**Table 5:** Binary classification results for each classifier (daily prediction).

Classifier	NN	SVM	*k*-NN	RF	GBM
**Sensitivity**	92.88%	77.96%	76.61%	88.47%	89.15%
**Specificity**	94.18%	90.91%	93.45%	94.54%	95.27%
**Accuracy**	93.51%	84.21%	84.73%	91.41%	92.1%

A second design is now considered, *i.e.* a longitudinal assessment. Therefore, two approaches were considered to test the performance of three classifiers with the highest accuracy (> 91%), *i.e.* NN, RF and GBM, on longer periods following a 5-day prediction. For both approaches, a daily feature extraction is required. Next, regarding the first approach referred to as “Averaging”, we averaged the features per 5-day (mean value of each consecutive 5 samples was computed), in a way that each sample represents the activity metric over 5 days instead of one. As for the second approach referred to as “Voting”, we kept the daily prediction and we selected the most frequently occurring prediction over 5 consecutive days. For example, if the classifier predicts four negatives and one positive, then the result over those 5 days is considered to be negative. The corresponding results for each approach are illustrated in Table 6.

**Table 6:** Binary classification results for three classifiers following a 5-day prediction.

Prediction	Averaging			Voting		
Classifier	RF	NN	GBM	RF	NN	GBM
**Sensitivity**	73.68%	78.95%	73.68%	96.49%	98.25%	91.23%
**Specificity**	96.15%	92.31%	96.15%	98.08%	98.08%	98.08%
**Accuracy**	84.4%	85.32%	84.4%	97.25%	98.17%	94.5%

The first technique (Averaging) leads to a high specificity at the expense of the sensitivity. As we can see, the sensitivity of each classifier is below 80%. It seems that a prediction based on the average of the features over longer periods destabilizes the classifier when it comes to the identification of physical impairment. Although highly specific systems are characterized by low false positive rates (and thus fewer number of false alarms), the ability to detect physical weakening is somewhat low in this case. The voting technique, contrarily to the averaging one, raises both the sensitivity and the specificity. The latter rose up to 98.08% for the three classifiers, meaning that one false alarm is generated every 8–9 months. Additionally, the sensitivity of NN is 98.25%, which makes it a reliable solution for this task.

### Discussion

This work has clarified some elements that were not well addressed in the literature, while interpreting different aspects of the physical impairment context. As mentioned previously, the study cohort includes elderly over 80 years of age who wore the sensing unit over relatively long periods during their daily routine in real world conditions.

On the one hand, the computed measurements are more meaningful in the analysis. For instance, it has been shown that the sedentary level differed significantly between frail and healthy subjects over 60 years of age when assessed over 48 hours [13]. However, this information is not sufficient in daily routine of octogenarians and older over longer periods, since our results showed that the activity rate is statistically not significant. While the evolution of this feature over time might be important for health monitoring, the value itself is unable to discriminate between both populations. Besides, vulnerable subjects were identified to be mostly lying when inactive [13] and to be sleeping longer [15] (a monitoring of 9.43 days on average). However, the sleep patterns do not distinguish frail from non-frail elderly according to our analysis. The sleep agitation, a novel characteristic in this context, does not seem to be well correlated with physical impairment, but is rather subject-dependent.

On the other hand, the proposed features can be computed automatically in unsupervised conditions while providing an accurate diagnosis. Indeed, a slower gait is an indicator to frailty [10]. Nonetheless, this measurement is usually evaluated under supervised conditions with a set of instructions. Feature *G*_5_ or *T*_*p*_, which is statistically significant, could replace gait velocity and identify older adults showing physical worsening.

Furthermore, the false alarm rate was somewhat large for daily prediction. It is worth noting that the frequency of diagnosis (between two decisions), a topic which received little attention in the literature, may affect the performance of systems. The proposed features are extracted over the course of the day, and so a long-term analysis seems more suitable for these types of features. According to the experimental results, a decision based on the most frequently occurring daily prediction over longer intervals (5 days for example), seems to be the best design for an alerting system targeting physical weakening. Features like the number of steps and EE for example possess high variability over small periods, *i.e.* they may differ considerably between consecutive days. This justifies the high performance, particularly for NN, when a longitudinal assessment was targeted. This novel set of features is able to separate both physical states (robust *vs* frail). Their evolution over time is a key to detect weakening from daily routine.

This work may serve as a basis for the paradigm of future monitoring systems, as seen in the prototype illustrated in S1 Fig. We developed a tool whose output is a daily report consisting of values and illustrations based on the proposed monitoring process. This application is firstly dedicated to the older adult, who would be the actor of his/her own health by having the necessary information on the evolution of his/her state of health if he/she wishes and on a voluntary basis. Moreover, this tool can also help the communication between patients and clinicians giving some objective measures that can help them in sharing information and diagnosis. These parameters are linked to the occurrence of an outcome of interest, like a certain disease and its association with risk factors. Rapid weakening, falling, consumption of medical care, hospitalization and other topics can motivate patients and their health professionals to adhere at continuous monitoring solution. The patients and health professionals staff can share, with the explicit agreement of the person and in respect of needed confidentiality, the evolution of these parameters and receive alarms in case of physical worsening. It might help the proposal of an intervention in view of supporting elderly people to live at home independently. This prototype, which is a handy tool, seems to be an answer to overcome these problems.

## Limitations and artifacts

The study cohort being somewhat small, our team is currently recruiting more participants to extend this study and thus generalize the results on a larger dataset. Moreover, some data were lost due to (i) lockdowns imposed by the French government (pandemic of covid-19), since the authorized team was not able to intervene and extract data, and (ii) some subjects forgetting to recharge their devices. This explains why the monitoring period varied between the participants.

Parameters like age, gender, and health status may affect the EE. However, applying a conventional MET value to all individuals of diverse profiles is a common practice [29]. In our study, we focused on the type and the intensity of movements. Even though the normalized EE might be over/under-estimated, the statistical significance and ability of *G*_9_ to separate both groups of elderly, as well as its evolution over time are what matter in detecting decline in physical capacities.

Finally, some artifacts are linked to the sensor which is worn under clothes. The measured temperature might be affected by the body temperature, but does not significantly affect *G*_7_ and *G*_8_ since we deal with difference in altitude. Besides, the sampling rate of the accelerometer and barometer might slightly change due to weather changes and differential-pressure (since long-term experiments were considered). Additionally, the internal clock of the micro-controller introduces some delay. These two phenomena might induce a certain shifting of maximum ±30 minutes to the 24-hour files. Consequently, some resultant files may be smaller/larger than 24 hours. Anyhow, the margin of error is acceptable.

## Conclusion

This paper presents a monitoring system based on a novel set of heterogeneous features extracted from acceleration and barometric signals. Contrary to the literature, a long-term clinical trial was done in free-living conditions, to investigate the utility of wearable sensors to detect physical weakening from daily routine. Both populations (non-frail and frail elderly) were characterized based on the computed measurements, and their data distribution was illustrated using PCA. It was demonstrated that these two populations are separable using non-linear boundaries and that a longitudinal assessment should be targeted using this type of measurements. The statistical significance of the proposed features as well as the efficacy of data-driven systems in this context were evaluated.

In addition to the extension of this pilot study, we plan to observe the evolution of this ensemble of features over time, during the transition from robustness to frailty, in order to assess frailty trajectories. Moreover, the study of local features linked to gait analysis is an ongoing research topic.

## Supporting information

S1 Fig Archetype for activity tracking. This daily report illustrates real data of an old person in free-living conditions. The goal of this graphical representation of these features is on the one hand, to provide necessary information on the evolution of health conditions for seniors who would become the actors of their own health, and on the other hand to help the communication between patients and clinicians. The first figure (blue bars) represents the activity rate (in %) during a specific day. The second row shows, on the one hand, the number of steps and the number of burned calories, and, on the other hand, the sleep pattern. Afterwards, the rate and duration of periodic movements are illustrated (third row). Finally, the localized moments during which the subject has used the lift/stairs are pictured in the last graph. Explicitly, the corresponding subject was highly active between 9 am and 12 pm. He was mostly inactive at night (while he was sleeping). Moreover, the sleep patterns are illustrated, where the cycles are pictured in green, and the interruptions in red. Two types of interruptions are detected: (i) small ones resulting from rotation or change of positions, and (ii) relatively long ones resulting from higher activity levels, where the subject is active and moving. The latter is represented by the second and fourth interruptions in the report, when the subject woke up and went to the toilet. This result is coherent with the activity rate diagram (blue bars), where it is shown that the subject was active between 1-2 am, and 4-5 am. Then he went into deep sleep again, since the fifth cycle is large. Furthermore, 3.3% of the subject’s activities were periodic between 11 am and 12 pm (orange bars). Meanwhile, his periodic movements never exceeded 25 minutes (yellow bars). Finally, the subject took the lift upward four times (one of them localized at 7:44 pm) and downward three times, and he descended the stairs at 12:13 pm. In our solution, 3 meters correspond to one floor.
(PDF)
