# Supplementary figures and images for: Data-driven systems to detect physical weakening from daily routine: A pilot study on elderly over 80 years old
Source: PLoS One. 2023 Jan 30;18(1):e0274306. doi: 10.1371/journal.pone.0274306 (PMC9886261; doi:10.1371/journal.pone.0274306)

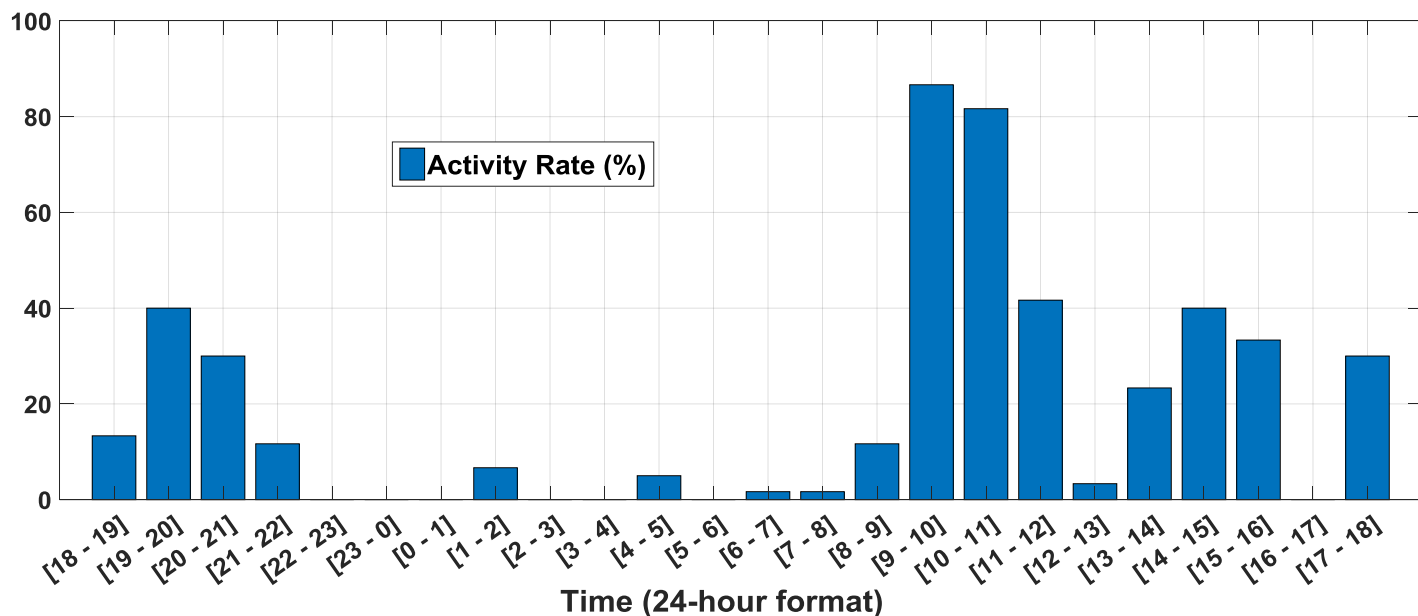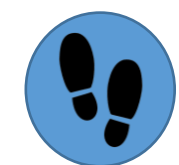

2653 steps

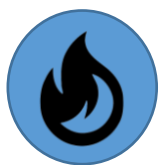

2416 kcal

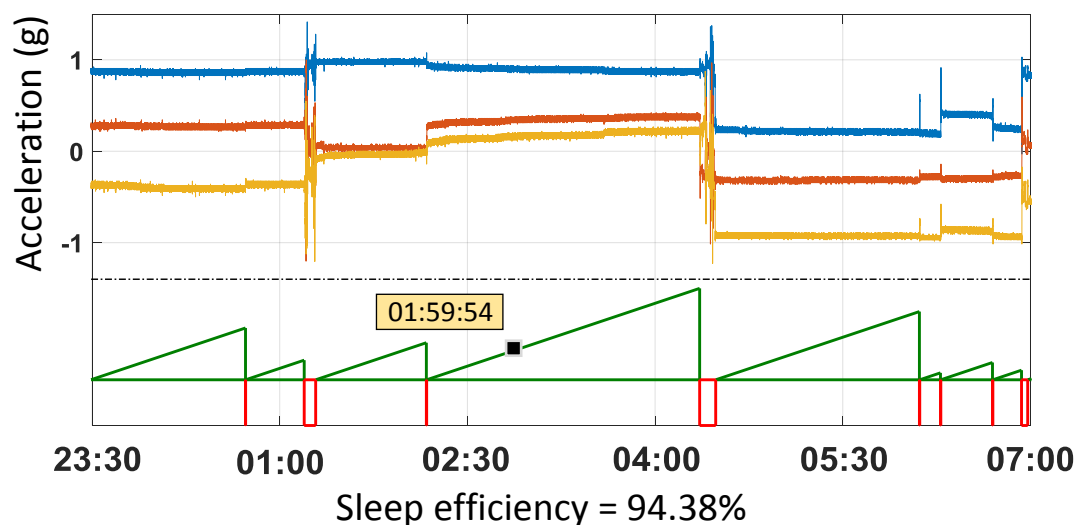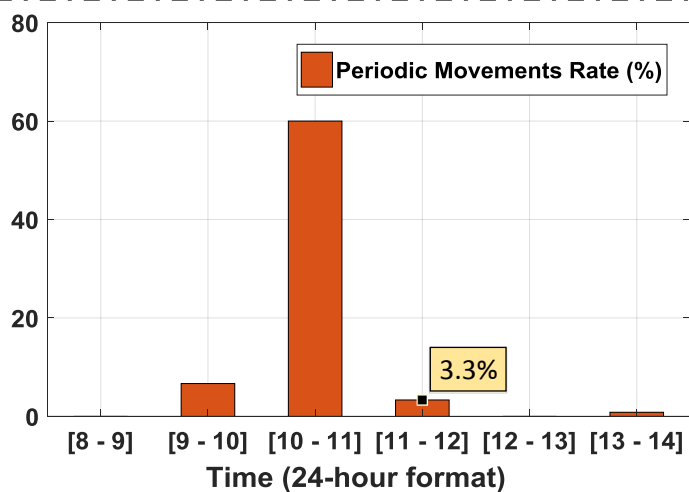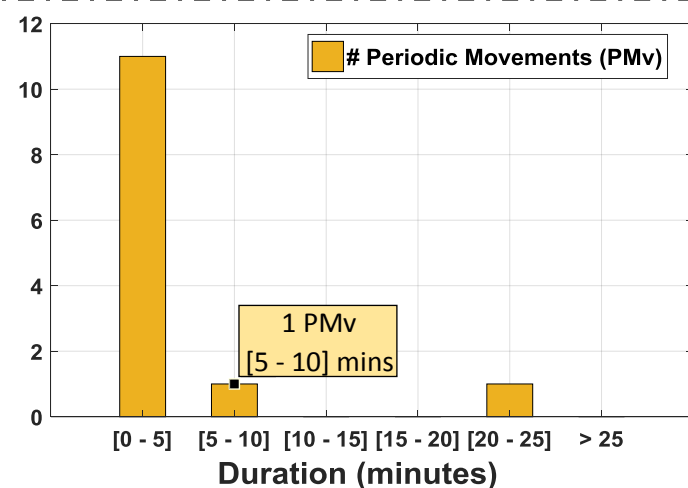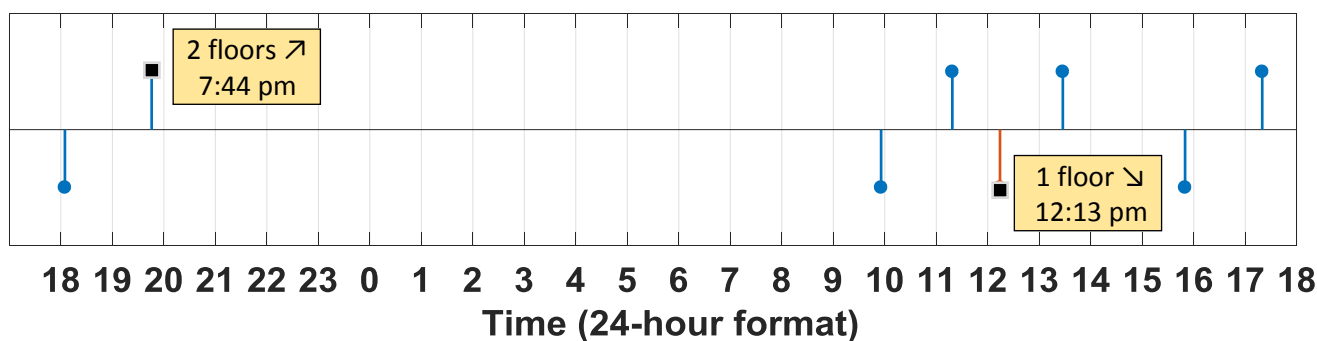

Supplement: S1 Fig — This daily report illustrates real data of an old person in free-living conditions. The goal of this graphical representation of these features is on the one hand, to provide necessary information on the evolution of health conditions for seniors who would become the actors of their own health, and on the other hand to help the communication between patients and clinicians. The first figure (blue bars) represents the activity rate (in %) during a specific day. The second row shows, on the one hand, the number of steps and the number of burned calories, and, on the other hand, the sleep pattern. Afterwards, the rate and duration of periodic movements are illustrated (third row). Finally, the localized moments during which the subject has used the lift/stairs are pictured in the last graph. Explicitly, the corresponding subject was highly active between 9 am and 12 pm. He was mostly inactive at night (while he was sleeping). Moreover, the sleep patterns are illustrated, where the cycles are pictured in green, and the interruptions in red. Two types of interruptions are detected: (i) small ones resulting from rotation or change of positions, and (ii) relatively long ones resulting from higher activity levels, where the subject is active and moving. The latter is represented by the second and fourth interruptions in the report, when the subject woke up and went to the toilet. This result is coherent with the activity rate diagram (blue bars), where it is shown that the subject was active between 1-2 am, and 4-5 am. Then he went into deep sleep again, since the fifth cycle is large. Furthermore, 3.3% of the subject’s activities were periodic between 11 am and 12 pm (orange bars). Meanwhile, his periodic movements never exceeded 25 minutes (yellow bars). Finally, the subject took the lift upward four times (one of them localized at 7:44 pm) and downward three times, and he descended the stairs at 12:13 pm. In our solution, 3 meters correspond to one floor. (PDF) [file pone.0274306.s001.pdf]
